# Longitudinal Estimated Glomerular Filtration Rate Trajectories in Children with Type 1 Diabetes

**DOI:** 10.1155/2023/6648920

**Published:** 2023-06-29

**Authors:** Kristen Favel, Cherry Mammen, Constadina Panagiotopoulos

**Affiliations:** ^1^Department of Pediatrics, University of British Columbia, Vancouver, British Columbia, Canada; ^2^Division of Nephrology, British Columbia Children's Hospital, Vancouver, British Columbia, Canada; ^3^Department of Pediatrics, University of California San Francisco, San Francisco, California, USA; ^4^Division of Nephrology, Benioff Children's Hospital, San Francisco, California, USA; ^5^Endocrinology & Diabetes Unit, British Columbia Children's Hospital, Vancouver, British Columbia, Canada

## Abstract

Although children with type 1 diabetes (T1D) are at risk for developing diabetic kidney disease (DKD), clinical practice guidelines do not uniformly recommend routine serum creatinine (SCr) monitoring, and data describing changes in renal function from diagnosis are lacking. As part of a quality improvement initiative, the Diabetes Clinic at British Columbia Children's Hospital in Vancouver, Canada, implemented routine serum SCr monitoring. This study describes estimated glomerular filtration rate (eGFR) trajectories and prevalence of decreased eGFR, hypertension, and albuminuria and their relationship to patterns of nephrology referral in a cohort of children aged ≤18 years (*n* = 307) with T1D recruited between December 2016 and February 2019. Annualized eGFR (ml/min/1.73 m^2^ per year) was calculated using the CKiD U25 formula and categorized as declining (<−3), stable (−3 to +3), and inclining (>+3). eGFR was categorized as normal (≥90), mildly decreased (60 to <90), and chronic kidney disease (CKD, <60). In this cohort, 54% were male; the median age at diagnosis and duration of T1D was 6.2 years and 6.9 years, respectively. Over a median follow-up of 2.3 years, declining, stable, and inclining trajectories were observed in 33%, 32%, and 35%, respectively. During their follow-up, 32% had mildly decreased eGFR, elevated blood pressures (≥90^th^ percentile), and/or abnormal urine albumin-creatinine ratios (≥2 mg/mmol), with <10% referred for nephrology assessment. Twenty-three percent of subjects had an eGFR <90; this subgroup was more highly represented in the declining trajectory group (vs. stable and inclining). Logistic regression analysis found female sex and higher baseline eGFR to be associated with a declining eGFR trajectory. In conclusion, these data challenge the commonly held paradigm that renal function remains stable in childhood T1D and supports systematic monitoring of renal function in children with T1D, as well as collaboration across disciplines, particularly endocrinology and nephrology, to provide evidence-based individualized care.

## 1. Introduction

Diabetic kidney disease, defined by the presence of albuminuria and/or reduced renal function, is a common complication of type 1 diabetes (T1D) [1, 2]. Though conventionally held to be an adult condition, a growing body of the literature has observed that DKD may be diagnosed in children with T1D [3–8]. Pediatric T1D guidelines (International Society for Pediatric and Adolescent Diabetes, American Diabetes Association, and Diabetes Canada) recommend screening for DKD with (1) blood pressure measurements at each routine clinic visit and (2) commencement of screening for albuminuria with an annual spot urine albumin-to-creatinine ratio (ACR) at puberty (or 10–12 years of age) and 2–5 years duration of T1D [9–11]. Notably, assessment of serum creatinine (SCr) or other measures of renal function are not uniformly recommended as part of standard pediatric T1D screening [9, 10] with only the International Society for Pediatric and Adolescent Diabetes including this recommendation recently in their 2022 guidelines [11]. Following recognition at our center that there was a high prevalence of acute kidney injury (AKI) in children with T1D presenting in diabetic ketoacidosis (DKA) without documented resolution upon hospital discharge, SCr was added to routine surveillance investigations as part of a quality improvement initiative [12]. An initial cross-sectional analysis of the SCr data revealed that more than 10% of children had decreased estimated glomerular filtration rate (eGFR) < 90 mL/min/1.73 m^2^. Furthermore, when analyzed cross-sectionally as a function of duration of T1D, eGFR declined by 1.4 mL/min/1.73 m^2^ per year [13].

Given the inextricable linkage of DKD to excess cardiovascular morbidity and mortality in adults with diabetes, early recognition of pediatric DKD is imperative [14, 15]. As highlighted by the 2014 report from the American Diabetes Association consensus conference, the pattern of development of DKD in children requires differentiation from the adult paradigm, and the trajectory of pediatric DKD from diagnosis needs to be elucidated in further detail [1]. Thus, in order to expand upon our cross-sectional eGFR findings, the primary objective of this study was to describe the longitudinal trajectory of the estimated glomerular filtration rate early in the course of pediatric T1D. As a secondary objective, we sought to describe the prevalence of the decreased glomerular filtration rate, hypertension, and albuminuria and their relationship to patterns of nephrology referral.

## 2. Materials and Methods

### 2.1. Study Population and Design

This ambidirectional cohort study, a study type which incorporates both retrospective and prospective data, was conducted at British Columbia's Children's Hospital (BCCH) in Vancouver, British Columbia (BC), Canada. Participants were identified and recruited through the BCCH T1D clinic between December 2016 and February 2019 and deemed eligible for inclusion if they were 18 years of age or younger and followed in the T1D clinic. Participants with a diagnosis of chronic kidney disease (CKD, defined as an eGFR <60 mL/min/1.73 m^2^) predating their diagnosis of T1D were excluded. As noted previously, SCr monitoring has been implemented for all children followed in the BCCH T1D clinic from the time of diagnosis of T1D, annually to biennially during childhood, up to and including their transition to adult care [13]. Participants consented to retrospective and prospective review of their medical records and were included in this analysis if at least 2 outpatient SCr measurements were completed by the time they were 18 years of age. All data collection occurred between December 2016 and December 2020. The University of British Columbia Clinical Research Ethics Board reviewed and approved the study protocol.

### 2.2. Data Collection and Case Definitions

A detailed description of data collection methods and case definitions utilized in our study has been previously published [13]. In brief, baseline demographic information collected included sex, age at diagnosis of T1D, and insulin modality. If subjects had an episode of DKA, inpatient information including date of DKA, height, and peak SCr values during admission was collected. Outpatient height, weight, systolic BP (SBP), and diastolic BP (DBP) values within 1 year of the date of each SCr value were collected. Resting SBP and DBP measurements were performed by the clinic nurse or physician, with elevated blood pressures repeated manually as per the 2017 American Academy of Pediatrics Clinical Practice Guidelines [16]. The body mass index (BMI) and BP percentiles were calculated using Canadian Pediatric Endocrine Group shiny applications, where BMI percentiles were based on the 2014 World Health Organization growth charts for Canada, published by the Public Health Agency of Canada, and BP percentiles were based on the 2017 American Academy of Pediatrics Clinical Practice Guidelines [16, 17].

All available outpatient SCr values for each study participant were collected and used to calculate eGFR. SCr was measured via enzymatic methods; laboratories in BC implemented SCr measurement standardization to the isotope dilution mass spectrometry (IDMS) reference method in 2004. All glycated hemoglobin (A1C) and urine ACR values within 1 year of the date of the SCr value were collected. Urine ACR was calculated from spot urine collections. Laboratories in BC determined urine albumin concentration by a radioimmunoassay, with urine ACR expressed as a ratio of the concentration of albumin (mg/L) to creatinine (mmol/L). As per the Diabetes Canada clinical practice guidelines, annual urine ACR tests were initiated for patients ≥12 years of age with >5 year duration of T1D [9]. Nephrology consultation was captured, including reason for referral, investigations completed, and interventions.

DKA was defined as per the International Society for Pediatric and Adolescent Diabetes guidelines [18]. AKI (in the context of an episode of DKA requiring admission to hospital) was defined and categorized as per the kidney disease improving global outcomes (KDIGO) SCr criteria (stages 1 to 3); urine output criteria were omitted as documentation of fluid balance data during DKA admissions was inconsistent across patient records [12, 19, 20]. An eGFR of 120 mL/min/1.73 m^2^ was used to back-calculate an expected baseline SCr level based on the chronic kidney disease in children formula [12]. The peak SCr value for each participant admitted with DKA was recorded for diagnosis and staging of AKI.

### 2.3. Renal Outcome Measures

Blood pressures were categorized by percentile as normal BP (less than the 90^th^ percentile), elevated BP (90^th^ to less than the 95^th^ percentile), or hypertension (at or above the 95^th^ percentile) based on the 2017 American Academy of Pediatrics Clinical Practice Guidelines [16]. Urine ACR values were categorized as abnormal at or above 2 mg/mmol after two consecutive measurements (with the first morning urine sample as per the clinic protocol for abnormal urine ACR) [21]. We calculated eGFR using the Bedside Schwartz equation (CKiD1), Chronic Kidney Disease in Children Under 25 equation (CKiD2, age- and sex-dependent; CKiD3, sex-dependent), and the European Kidney Function Consortium (EKFC) equation [22–24]. The CKiD2 equation was used for the analysis given (1) the accessibility to SCr measurements in outpatient laboratories in British Columbia, (2) the wide age span of our study subjects (from 2–18 years of age), and (3) its conservative estimate of eGFR trajectory as per the sensitivity analysis (Supplemental Figure 1).

The 2012 KDIGO clinical practice guidelines for the evaluation and management of CKD was used to categorize eGFR as normal (≥90 mL/min/1.73 m^2^), mildly decreased (60 to <90 mL/min/1.73 m^2^), and CKD (<60 mL/min/1.73 m^2^) [25]. The annualized change in eGFR (eGFR trajectory) was calculated as the difference between the first and last eGFR values divided by the time in years between the first and last eGFR values. eGFR trajectories were categorized as declining (<−3 ml/min/1.73 m^2^ per year), stable (−3 to +3 ml/min/1.73 m^2^ per year), and inclining (>+3 ml/min/1.73 m^2^ per year). A threshold value of 3 ml/min/1.73 m^2^/year was selected based on the literature describing the change in renal function in diabetes cohorts, with median rates of eGFR declining ranging from −1.5 to −4 ml/min/1.73 m^2^/year, where outside this threshold (inclining or declining trajectory) would signify an aberration from expected clinical trajectories [26–28]. As per KDIGO, CKD progression was defined as a decline in GFR category (≥90 to 60–89 ml/min/1.73 m^2^) accompanied by a 25% or greater drop in eGFR from the baseline [29].

### 2.4. Statistical Analysis

Descriptive data were reported as medians and interquartile ranges (IQR) and frequencies and percentages. Statistical comparisons were made with the Wilcoxon or Kruskal–Wallis test for nonnormally distributed continuous variables and Chi-square test for categorical variables. Univariate logistic regression (using stable eGFR trajectory as the reference category) was used to identify factors associated with having declining or inclining eGFR trajectories. Variables were selected a priori based on clinical importance, including sex and age at T1D diagnosis, as well as baseline clinical characteristics (BMI, BP, A1C, and eGFR) categorized using recognized cutoffs. In addition, BMI, BP, and A1C were categorized by average deviation from a recognized cutoff (85^th^ percentile for BMI, 90^th^ percentile for BP, and 9% for A1C) to account for change from baseline to follow-up and the burden of persistently elevated BMI, BP, and A1C. Variables that were significant at a *p* value less than 0.1 from univariate analysis were considered for the multivariate model; sex and age at diagnosis were included based on clinical significance. Effect measures were reported as the odds ratio (OR) and corresponding 95% confidence intervals (CI). For the eGFR equation sensitivity analysis, we compared the proportion of subjects with an eGFR <90 mL/min/1.73 m^2^ and the smoothed conditional mean plots of eGFR as function of time for the CKiD1, CKiD2, and CKiD3 and EKFC equations [22–24]. A *p* value less than 0.05 was considered significant. Statistical analyses were performed using R version 4.0.2 (R Foundation for Statistical Computing) [30].

## 3. Results

### 3.1. Study Population

In this analysis, 307 children with ≥2 eGFR values were included (203 with 2 eGFR values, 84 with 3 eGFR values, and 20 with 4 eGFR values). Baseline demographic and clinical data are described in Table 1. The median duration of follow-up for this cohort was 2.3 years (IQR 1.8–2.9), with a median duration of T1D of 4.5 years (IQR 2.0–7.5) at baseline. In the cohort, 54% were male. The median age at diagnosis of T1D was 6.3 years (IQR 3.6–9.5), with 78% diagnosed under 10 years of age. Insulin pump therapy was used by 43% of children, with the remainder using multiple daily subcutaneous insulin injections. Forty-one percent had a history of DKA, with 53% of subjects having concomitant AKI (42% stage 1, 42% stage 2, and 16% stage 3), see Supplemental Table 1 for description of subjects with DKA and stage 2-3 AKI. Forty-six percent of subjects had a BMI percentile at or above the 85^th^ percentile, and 28% had an A1C ≥ 9%.

### 3.2. Categorization of Trajectories Based on the eGFR Slope

**Figure 1:**
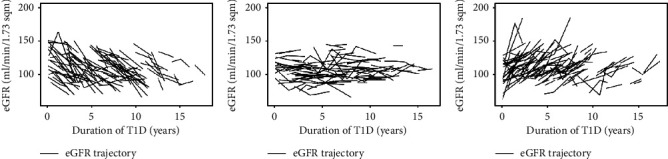
Categorization of trajectories based on the eGFR slope: (a) declining (*N* = 100); (b) stable (*N* = 99); (c) inclining (*N* = 108).

The median eGFR slope was +0.4 ml/min/1.73 m^2^ per year (IQR −5.0, +4.9). For females, the median eGFR slope was −0.8 ml/min/1.73 m^2^ per year (IQR −5.4, +4.4), and for males, it was +1.3 ml/min/1.73 m^2^ per year (IQR −4.7, +4.9) (*p* = 0.07). Categorized based on the eGFR slope, 33% had a declining trajectory, 32% had a stable trajectory, and 35% had an inclining trajectory, with the individual trajectories illustrated in Figures 1(a)–1(c), see Supplemental Figures 2A and 2B for eGFR trajectories of subjects with no history of DKA and history of DKA and stage 2-3 AKI, respectively. There were no significant differences between eGFR trajectory groups for the baseline demographic characteristics (Table 1; sex, age at diagnosis, and history of DKA or AKI). Subjects were of similar ages at eGFR at baseline and follow-up. Subjects in the inclining group had a shorter duration of T1D at baseline and follow-up than those in the declining and stable groups. The baseline eGFR was higher in the declining group than in the stable and inclining groups (Table 1).

From Table 1, BMI percentile increased from baseline to last follow-up in the declining and inclining groups, while remaining relatively unchanged in the stable group. SBP percentile increased across all groups. DBP percentile increased for the stable and inclining groups, while remaining relatively unchanged in the declining group. An increase in A1C from baseline to follow-up was noted in the inclining group, while A1C remained the same in the stable and declining groups.

### 3.3. Prevalence of Decreased eGFR, Hypertension, and Albuminuria

As an aggregate, the median eGFR for the cohort was 106 mL/min/1.73 m^2^ (IQR 96, 119). No study participant had an eGFR <60 ml/min/1.73 m^2^ at any point in their trajectory. Twenty-four percent of subjects had at least 1 eGFR value less than 90 ml/min/1.73 m^2^. Of the 18 subjects who had 2 or more eGFR values less than 90 ml/min/1.73 m^2^, 78% were female, and the median eGFR was 83 ml/min/1.73 m^2^ (IQR 76, 87). There was no significant difference in the number of subjects with an eGFR <90 by age (categorized as <10 years, 10–<13 years, and ≥13 years; *p*=0.53). From Table 2, there were a higher proportion of subjects with an eGFR less than 90 ml/min/1.73 m^2^ in the declining group than those in the stable and inclining groups. In total, 3% of subjects had CKD progression, with a median annualized change in eGFR of −10.6 ml/min/1.73 m^2^/year (IQR −13.9, −5.8) and a median follow-up eGFR of 74 ml/min/1.73 m^2^ (IQR 72, 75).

Overall, 16% and 4% of subjects had SBP and DBP measurements at or above the 95^th^ percentile, respectively. In the cohort, 183 subjects were eligible to begin urine ACR screening. Of the 132 subjects who completed 2 or more urine ACRs, 11% had two or more abnormal urine ACRs, with a median abnormal value of 5.2 mg/mmol (IQR 3.3, 12.7).

Overall, 32% (*n* = 98) of the cohort had a single abnormality or combination of abnormalities in BP, ACR, or eGFR, with only 9 subjects referred to nephrology. In this subcohort, 33% had a single abnormality (BP in 19/32, ACR in 13/32, and eGFR in 1/32) and 5% (*n* = 5) had a combination of abnormalities in BP, ACR, and eGFR. None of the subjects with CKD progression were referred to nephrology. All those referred were over 12 years of age and had diabetes for more than 5 years. Referrals were for AKI follow-up, hypertension, and albuminuria. Five subjects were started on angiotensin-converting enzyme inhibitors (ACEI; ramipril or enalapril) at a median T1D duration of 9.4 years (IQR 9.1–13.0).

### 3.4. Factors Associated with Declining and Inclining eGFR Trajectories

Table 3 highlights the univariate logistic regression of baseline demographic and clinical characteristics associated with declining and inclining eGFR trajectories in reference to a stable eGFR trajectory. Every 10 ml/min/1.73 m^2^ increase in baseline eGFR was associated with a 55% increase in the odds of a declining eGFR trajectory (*p* < 0.001). Supplemental Table 1 highlights the univariate logistic regression of longitudinal clinical characteristics (BMI, BP, and A1C) categorized by average deviation from recognized cutoffs.

Table 4 highlights the multivariate analysis of factors associated with a declining and inclining eGFR trajectory. Female sex was associated with 1.89 times higher odds of declining eGFR trajectory than males. For every 10 ml/min/1.73 m^2^ increase in the baseline eGFR, there was a 63% increase in the odds of a declining eGFR trajectory.

### 3.5. Comparison of Bedside Schwartz, CKiD U25, and EKFC Equations

The proportion of patients with an eGFR <90 mL/min/1.73 m^2^ was similar at 23%, 24%, and 21% using the CKiD1, CKiD2, and EKFC equations, respectively; it was higher at 32% using the CKiD3 equation. Supplemental Figure 1 illustrates the eGFR trajectories as a function of duration of T1D using the four equations.

## 4. Discussion

In this pediatric T1D cohort study, we identified two key findings. First, on repeated assessments of SCr, we observed that eGFR trajectories were heterogeneous, with 33% having a declining trajectory, 32% a stable trajectory, and 35% an inclining trajectory. Second, we observed that despite identification of abnormalities in BP, urine albumin excretion, and/or eGFR in 32% of the cohort, less than 10% of those subjects were referred to nephrology for more definitive diagnosis and management.

Repeated measures of SCr allowed for description of trajectories of renal function in this cohort, where there is a paucity of the literature describing the change in renal function over time in childhood. Pediatric data from the SEARCH cohort describes an eGFR decline in 23.8% and an increase in 2.8% of participants with type 1 diabetes [31]. In that cohort, baseline age was 11.4 years and the mean follow-up age was 17.9 years, where characteristics associated with an eGFR decline versus stable eGFR included male sex, younger age at diagnosis, higher baseline eGFR, and lower glucose level at the follow-up compared to the baseline visit [31]. Our study found a similar proportion of children and adolescents with a declining eGFR trajectory (32.5%), with a comparatively higher proportion with an inclining eGFR trajectory (35.2% vs. 2.8%). This may be reflective of a higher proportion of repeated eGFR measures earlier postdiagnosis in our cohort, where the inclining eGFR trajectory group had a shorter duration of T1D than the stable or declining eGFR trajectory groups. This may also reflect variation in eGFR across equations, where the cystatin-C-basedCKiDCr-CysC equation was used in the SEARCH analysis.

The heterogeneity in the eGFR trajectory reported in this study contrasts with longitudinal studies of healthy children, in which GFR has been described as linear and stable over childhood; in contrast, our findings are more in keeping with the adult diabetes literature which has demonstrated variable rates of decline in renal function with time, ranging from −0.4 to −72 mL/min/1.73 m^2^ per year in adults who with normal to advanced CKD, respectively [32–34]. Our study also found that higher baseline eGFR, which may also be referred to as hyperfiltration, was highly associated with the eGFR trajectory. Although the relationship between hyperfiltration and declining GFR has been documented in adult T1D and other pediatric CKD cohorts, application of the concept in pediatric clinical care and research is hindered by a lack of consensus of its definition [35–38]. We also considered baseline AKI in our analysis; however, having a single episode of AKI was not strongly associated with the eGFR trajectory. This association may not have been identified given the small sample size for severe AKI (*n* = 39). In addition, subjects with repeated episodes of DKA and AKI were not characterized, and these individuals may be at higher risk for CKD and merit further longitudinal study.

In contrast to the SEARCH cohort, we found that female sex was more highly associated with having a declining eGFR trajectory, and this substantiated the findings of our original cross-sectional analysis [13]. Sex-related differences in renal outcomes in T1D have been studied extensively in adults with variable results depending on the cohort and study era, with sex-hormone levels, kidney hemodynamics, adiponectin concentrations, oxidative stress, and water-electrolyte homeostasis postulated as potential reasons for differences in a risk for DKD development and progression; however, there are limited data investigating the sex-related differences in renal function trajectories in children and adolescents [39–41]. This was addressed in the CKiD cohort which found a faster progression of CKD in females with glomerular diseases, but no significant sex difference was found in those with nonglomerular diseases, perhaps explained by sex differences in underlying primary kidney disease and in the baseline GFR [36].

The findings in this study support the growing body of evidence that DKD is demonstratable during childhood T1D. The persistent blood pressure abnormalities noted in our cohort are comparable to reported prevalence of hypertension of 6–16% of children with T1D and elevated in comparison to the prevalence of hypertension in the general pediatric population (approximately 4%) [42–45]. In addition, the prevalence of albuminuria in our cohort was comparable to that in other T1D cohorts and registries, ranging from 4–8% [7, 46, 47]. With the added granularity of renal function, we were able to ascertain the degree of overlap in decreased renal function and abnormalities in BP and urine ACR. Our study identified that only a small proportion of children and adolescents were referred to nephrology for further assessment. While this may reflect practice at a single centre, nephrology referral patterns have not been elucidated in detail in the pediatric T1D literature. One possible reason for the limited number of referrals at our centre is that endocrinologists may not be recognizing that SCr or eGFR is abnormal because most Canadian laboratories do not provide automated systems for eGFR calculation using an up-to-date height. In addition, we recently published a study showing suboptimal adherence to albuminuria screening at our centre, thus further limiting DKD recognition [48]. Improving the recognition of albuminuria, abnormal eGFR, and abnormal BP is critical, given our findings that only subjects referred to nephrology were initiated on BP-lowering medications. As well, other pediatric T1D cohorts have noted undertreatment of hypertension and albuminuria [49–53]. Reassuringly, indications for referral to a pediatric nephrologist have been added to the most recent iteration of the ISPAD guidelines; however, reasons for nonreferral or undertreatment of DKD need to be delineated to better support clinicians and strengthen current clinical practice guidelines [11].

The strengths of this study include the use of repeat measures of BP, urine ACR, and eGFR to quantify renal outcomes and trajectories of renal function in a large sample of children and adolescents over a wide time frame, from less than 1 year to 15 year duration of T1D. Challenges inherent to this area of study include the lack of a consensus on the optimal approach for assessment of renal function in children and adolescents with and without T1D [54–59]. While measured GFR remains the gold standard for assessment of renal function, it may be cumbersome for routine application in the clinical setting and may require customized measurement protocols based on the anticipated level of GFR, as compared to equations utilizing SCr and cystatin-C (CysC) [60]. Although eGFR equations utilizing Cys-C may be advantageous in children and adolescents with higher or lower than expected muscle mass for their height, it remains limited in its clinical accessibility and affordability. Specifically, studies have indicated that CysC is not widely available across regions (only 67% of respondents reporting access to CysC as per the International Society of Nephrology Global Kidney Health Atlas survey) [61]. In addition, CysC reagents remain more expensive than SCr, with varying guidelines for testing reimbursement (typically relegated for individuals with an SCr-based eGFR <60) [61, 62]. While our study indicates that CKiD2 (CKiD under 25 and age- and sex-dependent) provides the most conservative estimate of the eGFR trajectory compared to other equations analyzed, more longitudinal research and collaboration across disciplines are required to ascertain how renal function may be integrated into the clinical practice and pediatric T1D guidelines.

Limitations to this study include the lack of data pertaining to pubertal status for adolescents, as Tanner staging is infrequently performed in our outpatient T1D clinic. As GFR may change in relation to sex-hormone levels, these data emphasize the need for endocrinologists to assess pubertal status regularly [39, 40]. In addition, it was outside the scope of our study to characterize subjects with multiple episodes of DKA, and in a previous publication, we found that the repeated episodes of DKA occurred very infrequently (∼7.5 cases per year) [12]. Given that DKA and associated AKI may have deleterious impacts on renal function, future prospective studies with larger sample sizes are needed to further understand this risk factor. Lastly, while we were able to document changes in eGFR on repeated measures over a broad timespan, further prospective studies evaluating renal complications of T1D in individuals from diagnosis to the transition to adult care will be needed to better understand this unique population.

## 5. Conclusions

In conclusion, this study demonstrates that renal complications are common in children with T1D, including persistently decreased renal function in combination with albuminuria and hypertension. These data challenge the commonly held paradigm that renal function remains stable in childhood, with a significant proportion of our cohort demonstrating a declining eGFR trajectory. These data support the systematic monitoring of BP, urine albumin excretion, and renal function in children with T1D, as well as collaboration across disciplines, particularly endocrinology and nephrology, to implement systems for providing evidence-based, individualized care. Given the implications of similar findings in adult cohorts, further longitudinal study of renal function in T1D from childhood through adulthood including a larger sample size is warranted, with the goal of implementing interventions to halt the progression of DKD in this population.

## Figures and Tables

**Table 1:** Baseline and follow-up characteristics stratified by eGFR trajectory category.

	All	Declining	Stable	Inclining
	(*N* = 307)	(*N* = 100)	(*N* = 99)	(*N* = 108)
*Baseline demographic characteristics*				
Male sex	165 (54%)	46 (46%)	55 (56%)	64 (59%)
Age at diagnosis (years)	6.2 (3.6, 9.5)	6.7 (4.2, 9.3)	5.8 (3.3, 9.5)	5.7 (3.5, 9.6)
History of DKA and AKI	67 (22%)	20 (30%)	25 (37%)	22 (33%)

*Baseline clinical characteristics*				
Age at eGFR (years)	12.3 (8.5, 14.8)	12.7 (9.3, 15.3)	12.0 (8.6, 14.3)	12.2 (7.6, 15.4)
Duration of T1D (years)	4.5 (2.0, 7.5)	4.8 (2.2, 8.0)	4.7 (2.3, 7.4)	3.7 (0.9, 6.7)
eGFR (ml/min/1.73 m^2^)^a^	106 (96, 119)	116 (105, 129)	104 (96, 116)	101 (93, 111)
BMI percentile	74 (51, 92)	70 (47, 92)	71 (55, 89)	80 (50, 92)
SBP percentile	55 (38, 75)	55 (43, 76)	49 (29, 71)	59 (40, 74)
DBP percentile	49 (34, 64)	49 (33, 64)	45 (35, 63)	51 (35, 64)
A1C (%)	7.8 (7.3, 8.6)	7.9 (7.4, 8.7)	7.8 (7.4, 8.7)	7.8 (7.2, 8.5)

*Follow-up clinical characteristics*				
Age at eGFR (years)	14.9 (10.9, 17.2)	15.6 (12.0, 17.5)	14.6 (10.9, 16.5)	14.7 (10.3, 17.4)
Duration of T1D (years)	6.9 (4.0, 9.9)	7.6 (4.0, 10.4)	7.2 (4.6, 10.0)	5.8 (3.5, 9.1)
eGFR (ml/min/1.73 m^2^)^b^	108 (95, 119)	95 (88, 109)	104 (96, 113)	118 (108, 132)
BMI percentile^e^	75 (48, 91)	78 (53, 93)	72 (47, 93)	74 (46, 90)
SBP percentile^c,d,e^	77 (58, 89)	76 (56, 88)	76 (57, 90)	79 (62, 88)
DBP percentile^d^	52 (38, 72)	47 (32, 67)	56 (39, 72)	55 (39, 76)
A1C (%)^e^	7.9 (7.2, 8.7)	7.9 (7.1, 8.5)	7.8 (7.3, 8.7)	8.0 (7.2, 9.0)

Median (IQR) reported for quantitative variables and absolute (%) for qualitative variables. ^a^Kruskal–Wallis *p* value <0.05 for baseline comparison. ^b^Kruskal–Wallis *p* value <0.05 for follow-up comparison. ^c^Wilcoxon *p* value <0.05 for declining trajectory comparison from baseline to follow-up. ^d^Wilcoxon *p* value <0.05 for stable trajectory comparison from baseline to follow-up. ^e^Wilcoxon *p* value <0.05 for inclining trajectory comparison from baseline to follow-up.

**Table 2:** Clinical characteristics stratified by eGFR trajectory category.

Clinical characteristics	All	Declining	Stable	Inclining
	(*N* = 307)	(*N* = 100)	(*N* = 99)	(*N* = 108)
eGFR <90 ml/min/1.73 m^2a^	73 (24%)	35 (48%)	17 (23%)	21 (29%)
BMI ≥85^th^ percentile	140 (46%)	49 (35%)	40 (28%)	51 (36%)
SBP ≥90^th^ percentile	90 (29%)	27 (30%)	31 (34%)	32 (36%)
DBP ≥90^th^ percentile	33 (11%)	9 (27%)	8 (24%)	11 (33%)
A1C ≥ 9%	81 (26%)	25 (31%)	24 (30%)	32 (38%)
ACR ≥2 mg/mmol^b^	14 (11%)	4 (6%)	9 (16%)	1 (2%)

^a^ *p* value <0.05. ^b^Total of 132 subjects eligible for ACR screening (47 in declining, 38 in stable, and 47 in inclining categories).

**Table 3:** Baseline demographic and clinical characteristics associated with nonstable eGFR trajectories.

Baseline characteristics	OR (95% CI)	
	Declining eGFR trajectory^a^	Inclining eGFR trajectory^a^
Male sex	0.68 (0.39, 1.19)	1.16 (0.67, 2.02)
*Age at diagnosis*		
5–<10 years	1.43 (0.76, 2.68)	1.08 (0.58, 2.00)
≥10 years	1.61 (0.75, 3.47)	1.48 (0.71, 3.11)
History of DKA	0.67 (0.38, 1.19)	1.00 (0.58, 1.73)
History of AKI	0.74 (0.38, 1.44)	0.76 (0.39, 1.45)
*BMI percentile*		
50–<85^th^	0.54 (0.26, 1.11)	0.54 (0.26, 1.11)
≥85^th^	0.91 (0.43, 1.92)	1.10 (0.52, 2.29)
*SBP percentile*		
50–<90^th^	1.48 (0.83, 2.64)	1.72 (0.97, 3.06)
≥90^th^	1.25 (0.33, 4.79)	2.31 (0.74, 8.03)
*DBP percentile*		
50–<90^th^	1.10 (0.62, 1.96)	1.26 (0.72, 2.21)
≥90^th^	1.62 (0.26, 12.7)	2.64 (0.54, 19.0)
eGFR (ml/min/1.73 m^2^)	1.04 (1.03, 1.07)	0.98 (0.96, 0.99)
A1C (%)	1.09 (0.86, 1.38)	0.98 (0.77, 1.25)
ACR ≥2 mg/mmol^b^	1.01 (0.48, 2.14)	0.61 (0.27, 1.36)

^a^Reference category: stable eGFR trajectory. ^b^Based on the ACR-eligible cohort.

**Table 4:** Baseline demographic and clinical characteristics associated with a declining and inclining eGFR trajectory.

Baseline characteristics	Odds ratio (95% CI)	
	Declining eGFR trajectory^a^	Inclining eGFR trajectory^a^
Male sex	0.53 (0.28, 0.97)	1.30 (0.73, 2.32)
*Age at diagnosis*		
5–<10 years	1.27 (0.64, 2.49)	1.13 (0.61, 2.13)
≥10 years	1.82 (0.80, 4.17)	1.51 (0.72, 3.24)
eGFR (ml/min/1.73 m^2^)	1.05 (1.03, 1.07)	0.98 (0.96, 0.99)
A1C (%)	0.96 (0.74, 1.25)	1.01 (0.79, 1.30)

^a^Reference category: stable eGFR trajectory.

## Data Availability

The data used to support the findings of this study are available from the corresponding author upon request.

## Supplementary Materials

Supplementary Materials Supplemental Figure 1: smoothed conditional means plot of eGFR with duration of T1D using the Bedside Schwartz (CKiD1), Chronic Kidney Disease in Children Under 25 equation (CKiD2, age- and sex-dependent), Chronic Kidney Disease in Children Under 25 equation (CKiD3, sex-dependent), and the European Kidney Function Consortium (EKFC) equations. Supplemental Table 1: Cinical characteristics of subjects with concurrent DKA and stage 2-3 AKI (*N* = 39). Supplemental Figure 2A-B: Categorization of trajectories based on DKA/AKI status. Supplemental Table 2: Longitudinal clinical characteristics associated with nonstable eGFR trajectories.
