# Supplementary material for: Longitudinal Estimated Glomerular Filtration Rate Trajectories in Children with Type 1 Diabetes
Source: Pediatr Diabetes. 2023 Jun 29;2023:6648920. doi: 10.1155/2023/6648920 (PMC12017176; doi:10.1155/2023/6648920)
Supplement: Supplementary Materials — Supplemental Figure 1: smoothed conditional means plot of eGFR with duration of T1D using the Bedside Schwartz (CKiD1), Chronic Kidney Disease in Children Under 25 equation (CKiD2, age- and sex-dependent), Chronic Kidney Disease in Children Under 25 equation (CKiD3, sex-dependent), and the European Kidney Function Consortium (EKFC) equations. Supplemental Table 1: Cinical characteristics of subjects with concurrent DKA and stage 2-3 AKI (N = 39). Supplemental Figure 2A-B: Categorization of trajectories based on DKA/AKI status. Supplemental Table 2: Longitudinal clinical characteristics associated with nonstable eGFR trajectories. [file 6648920.f1.docx]

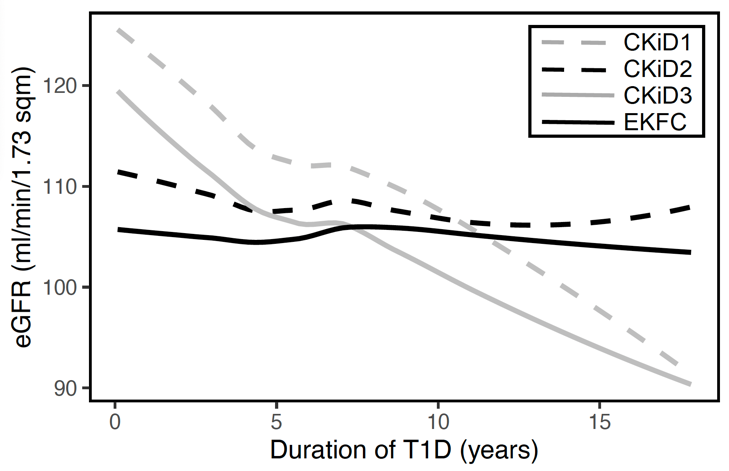


**Supplemental Figure 1.** Smoothed conditional means plot of eGFR with duration of T1D using the Bedside Schwartz (CKiD1), Chronic Kidney Disease in Children Under 25 equation (CKiD2, age- and sex-dependent), Chronic Kidney Disease in Children Under 25 equation (CKiD3, sex-dependent) and the European Kidney Function Consortium (EKFC) equations

| **Supplemental Table 1.** Clinical characteristics of subjects with concurrent DKA and stage 2-3 AKI (N=39) | |
| --- | --- |
| Male sex | 18 (46%) |
| Age at diagnosis (years)  <5 years  5-<10 years  $\geq$10 years | 5.6 (1.6, 10.6)  18 (46%)  10 (26%)  11 (28%) |
| eGFR slope (ml/min/1.73 m^2^)  Declining  Stable  Inclining | -0.5 (-3.6, +5.0)  12 (31%)  14 (36%)  13 (33%) |
| eGFR <90 ml/min/1.73 m^2^ | 2 (5%) |
| BMI $\geq$85^th^ percentile | 14 (36%) |
| SBP $\geq$90^th^ percentile | 4 (10%) |
| DBP $\geq$90^th^ percentile | 2 (5%) |
| A1C $\geq$9% | 8 (20%) |
| ACR $\geq$2 mg/mmol ^a^ | 1 (8%) |
| ^a^ Of the 17 subjects eligible for ACR screening, 12 had $\geq$2 ACRs | |

**
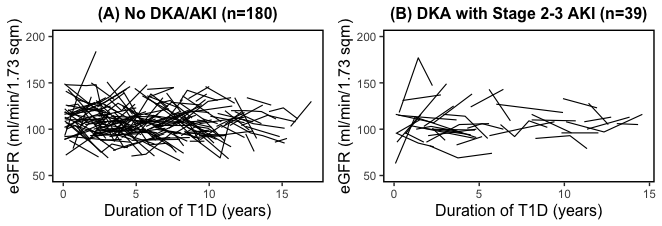
**

**Supplemental Figure 2A-B.** Categorization of trajectories based on DKA/AKI status

| **Supplemental Table 2.** Longitudinal clinical characteristics associated with non-stable eGFR trajectories | | |
| --- | --- | --- |
| Characteristics | OR (95% CI) | |
|  | Declining eGFR trajectory ^a^ | Inclining eGFR trajectory ^a^ |
| BMI $\geq$85^th^ percentile | 1.08 (0.59, 1.95) | 1.14 (0.64, 2.03) |
| SBP $\geq$90^th^ percentile | 1.30 (0.46, 3.78) | 0.77 (0.24, 2.41) |
| DBP $\geq$90^th^ percentile | 1.50 (0.24, 11.6) | 0.91 (0.11, 7.75) |
| A1C $\geq$9% | 1.09 (0.86, 1.38) | 0.98 (0.77, 1.25) |
| ^a^ Reference category: Stable eGFR trajectory | | |
